# Le don d´organes en Algérie: limites et perspectives

**DOI:** 10.11604/pamj.2021.39.232.30716

**Published:** 2021-08-10

**Authors:** Yassin Rekhif

**Affiliations:** 1 Faculté de Médecine d´Alger, Conseil Scientifique de l´Agence Nationale des Greffes, Alger, Algérie

**Keywords:** Don d´organes, transplantation, greffons, donneurs vivants, don croisé, donneurs décédés, Organ donations, transplantation, transplanted organs, living donors, cross-border donation, deceased donors

## Abstract

Le but de cet article est de mettre en exergue la nécessité et l´obligation d´associer au don d´organes provenant des vivants, celui issu des personnes décédées, une source de greffon dont l´apport est inégalable, un plaidoyer avec un triple objectif: 1) analyser l´apport du don issu des vivants, en termes de chiffre, à travers deux éléments: le degré d´implication de nos compétences médico-chirurgicales et nos dispositions réglementaires qui lui sont relatives. 2) Montrer que, ce don issu des vivants, même optimisé, restera toujours en deçà des besoins de nos malades et il n´apporte qu´une partie de la solution. 3) L´autre partie se trouve dans les services de réanimation, le donneur décédé, une source de don qui ne peut être substituée par celle des vivants et qui concerne nos malades ne disposant d'aucun donneur vivant et surtout ceux en attente d´un organe vital: cœur, poumon, foie, ou un rein pour ceux n'ayant plus la possibilité de faire la dialyse. Des patients condamnés à mourir s´ils ne sont pas transplantés dans les semaines à venir. A cet égard, seuls le professionnalisme et l´entière disponibilité du personnel dédié à cette activité, sont capables de nous débarrasser des préjugés qui accablent, à ce jour, injustement et systématiquement l´implication de notre société vis-à-vis de ce type de don, salvateur. Les pays ayant su promouvoir et se servir de cette source de don, un programme national lui a été priorisé et déployé en réseaux. Un programme aux résultats sûrs, fondé sur des normes universelles d´éthique de recrutement, de formation et organisationnelles. Pour les autorités sanitaires de ces mêmes pays, il est irrationnel et déraisonnable que ces greffons soient partageables avec les malades des autres pays: tant que cette source de don demeure l´unique à permettre la seule modalité thérapeutique salvatrice pour ce type de malades graves et tant que ce type de greffons resteront insuffisants pour satisfaire les besoins de tous leurs patients.

## Abstract (en)

The purpose of this study is to highlight the need and the obligation to combine living donation with deceased one, this later is an incomparable source of organ donation, a plea with a triple objective: 1) analyzing the contribution of living donations in terms of numbers, through two elements: the degree of involvement of our medico-surgical competencies and our regulations governing organ donations. 2) Showing that, even though optimized, living donations will always fall short of the needs of our patients and are only part of the solution. 3) The other part is deceased donors: a source of donation which can not be substituted by that of the living donors, and which concerns patients awaiting a vital organ with no possibility of another substitute treatment. Patients are sentenced to death if not transplanted in a few weeks. In this respect, only professionalism and full staff availability can rid us of prejudices which unjustly and systematically overwhelm our society with regard to this type of life-saving donations. In countries promoting and performing this source of donation, a national priority planning network has been developed. This program ensures reliable results, based on universal standards for ethics, recruitment, training and organization. For health authorities of these same countries, the sharing of these organs with others countries is irrational and unreasonable. As long as these organs remain the only life-saving for this type of seriously ill patients, and this source of grafts will be insufficient to meet the needs of all their patients.

## Perspectives

### Introduction

En Algérie, l´activité de transplantation d´organes, à ce jour, se limite à la greffe rénale et à celle du foie. Deux activités qui reposent casi-exclusivement sur une seule source: les donneurs vivants. On estime le nombre de malades insuffisants rénaux chroniques à environ 25 000, parmi 43 millions habitants, dont 1500 sont éligibles à une transplantation, une estimation annuelle rendue possible grâce à la dialyse et aux données statistiques émanant de nos centres de dialyse. Un besoin annuel minimal donc de 35 greffes rénales pmh, pour un ratio de greffes réalisées qui stagne entre 6,5 et 7 par million d´habitants (pmh) (aux alentours de 270 greffes rénales/an), depuis environ 5 ans [1]. Concernant les autres patients, au stade terminal de leurs insuffisances hépatique, cardiaque ou pulmonaire, il est difficile d´avoir des estimations annuelles fiables de leurs nombres et surtout de ceux d´entre eux qui sont éligibles à une transplantation, une difficulté liée essentiellement à l´absence d´un organe artificiel de suppléance pour ce type de patients. Par ailleurs le taux annuel de transplantations hépatiques réalisées depuis environ 5 ans, n´a jamais pu atteindre 0,3 greffes pmh [1]. Pour toutes ces raisons, les arguments apportés dans cette contribution, se référeront essentiellement aux données de l´activité de greffe rénale, aux besoins de nos malades en matière de transplantation d´organes et en partie aux données rapportées par la littérature.

### Le don d´organes entre les vivants en Algérie

#### Degré d´implication de nos compétences médico-chirurgicales concernant la greffe rénale

Ce chiffre annuel d´environ 7 transplantations rénales pmh réalisé à partir uniquement des donneurs vivants, ne serait jamais atteint, sans l´implication active des compétences médico-chirurgicales de nos établissements hospitalo-universitaires. Et pour mieux optimiser cette activité, ces équipes continuent à pousser les limites des indications de ce type de don, comme pour les donneurs dont les receveurs présentent des donor specific antibodies (DSA), les donneurs hypertendus bien équilibrés avec juste une monothérapie, les donneurs âgés avec une clairance rénale isotopique satisfaisante, ou ceux techniquement plus compliqués, dont le bilan d´évaluation chirurgical prévoit des risques et des difficultés de réimplantation vasculaire ou urologique du futur greffon. Parmi ces difficultés, en plus des variations anatomiques tel que la multiplicité artérielle et la duplicité urétérale, ou un rein mal roté avec un bassinet anormalement situé, il y´a celles liées à la présence d´une lésion rénale unilatérale, isolée et qui soit sans conséquence fonctionnelle, comme une lithiase pyélique, un syndrome de jonction pyélo-uretèrale, une plaque d´athérome ou une dysplasie fibromusculaire artérielle limitées sur l´artère du futur greffon, des lésions qu´il faut évidemment corriger juste après le prélèvement. Pour ces receveurs et à défaut de leurs trouver d´autres donneurs qui soient médico-chirurgicalement plus simples, nos équipes hospitalo-universitaires n´hésitent pas à prendre des risques mais mesurés, avec cette catégorie de donneurs, préservant ainsi à ces patients leur unique chance d´être transplanté. Et malgré cette persévérance pour optimiser l´apport de cette source de don, il arrive à nos équipes, de ne pas greffer pendant deux semaines, voire plus, par manque de donneurs.

***Nos dispositions réglementaires régissant ce type de don sont-elles assez permissives?*** Il est essentiel, avant de répondre à cette question, de rappeler que le donneur vivant, cet individu indemne de toute pathologie qui, pour venir en aide à son proche malade en lui offrant une survie et une qualité de vie meilleures et à notre système de santé un rapport coût/ efficacité incomparable à celui de la dialyse, s´expose à des risques de morbimortalité. Et de rappeler aussi que ce type de don soulève des inquiétudes, à savoir le trafic d'organes, une éventuelle pression exercée par la famille sur l´un de ses membres pour l´obliger à faire don d´un organe ou un conflit de missions lié aux deux responsabilités exercées simultanément par ces équipes de greffes: celle d´assurer l´intérêt de leur activité de greffe en termes de chiffres et celle d´assurer la sécurité de ce donneur. D´une part, face à un tel geste d´une générosité inégalée et un tel sacrifice, d´une grande utilité pour la santé publique, la logique veut que nos textes de lois relatives à ce type de don soient les plus permissifs. D´autre part, face à ces éventuelles dérives et aux risques de morbimortalité, la logique veut également que ces mêmes dispositions réglementaires soient à la fois dissuasives et restrictives. Concernant nos textes de loi, dont les dernières modifications ont été apportées il y´a 3 ans: nous pensons qu´elles évoluent parallèlement à l´évolution de notre société et à celle des connaissances liées à cette activité d´une part et d´autre part qu´elles ont toujours adhéré à ces deux lignes directrices qui paraissent contradictoires mais qui en fait ne sont que complémentaires: à la fois des dispositions incitatives au don et d´autres protectrices de ce donneur vivant.

### Notre loi et le cercle des personnes vivantes autorisées au don d´organes

**Le cercle familial** autorisé au don s´étend jusqu´aux membres de la famille du troisième degré, ce cercle qui s´est également élargit au don entre époux.

**Le don croisé:** consiste à récupérer deux couples de donneurs-receveurs, où chacun des deux donneurs présente une incompatibilité avec son receveur apparenté, puis s´arranger à ce que le donneur du couple A puisse donner son rein au receveur de l´autre couple B avec qui il n´a aucun lien de parenté et aucune contre-indication et réciproquement. Bien qu´il s´agisse d´un don extra familial, le don croisé a été introduit dans notre récente loi de santé, parce qu´il offre 4 avantages de taille: 1) un risque de marchandisation d´organes très infime par rapport aux autres types de don non apparentés, un risque identique à celui du don intrafamilial. 2) L´augmentation du nombre des donneurs vivants, par la récupération de ceux ayant été définitivement récusés juste pour une contre-indication immunologique absolue. 3) Des résultats en termes de survie du greffon à moyens et à long terme, de loin supérieurs à ceux d´un don apparenté comportant un risque de conflits immunologiques très élevé. 4) Et un coût nettement plus faible par rapport aux greffes dont le statut immunologique exige en pré-greffe des protocoles couteux de désimmunisation associant des techniques d´épuration des DSA et de single antigen pour le suivi de leur *median fluorescence intensity* (MFI) en plus d´un anticorps monoclonal anti CD20.

**Le don affectif:** entre personnes ayant une relation affective en dehors de tout lien familial: beaucoup de pays, pour éviter le risque d´une décision prise sous l´emprise d´une impulsion passagère et aussi pour dissuader tout développement d´une fausse liaison motivée par une intention frauduleuse, exigent une durée de vie commune suffisamment longue pour témoigner du caractère « étroit et stable » de cette liaison affective. Or dans les sociétés arabo-berbère-musulmanes et notamment celles du Maghreb, la majorité de leurs populations gardent un regard septique sur ce type de relations, qui de surcroit demeurent très marginales en termes de nombre, rendant ainsi ces sociétés non encore prêtes pour débattre et envisager ce type de don, qui risque pour le moment de créer plus de problèmes que d´en résoudre un.

**Le don altruiste:** un autre type de don non apparenté, lorsqu'une personne se propose spontanément pour donner un de ses reins à un candidat en attente de greffe: tant que les prérequis organisationnels indispensables à l´activité de greffe à partir de donneur décédé n´ont pas été encore installés dans notre pays, ce don ne pourrait ni être envisagé ni autorisé, puisque le don altruiste doit être à la fois: non dirigé pour éviter toute suspicion de transaction commerciale en pré don et aussi anonyme pour éviter tout chantage financier ultérieure, en post don. Une attribution qui exige donc et comme pour celle des greffons issus des donneurs décédés, une liste nationale actualisée et informatisée des candidats inscrits à la greffe, associée à un système informatique national d´attribution de ces greffons, pour assurer à la fois l´anonymisation de ce donneur altruiste et l´équité et la transparence nécessaires à cette attribution.

### Notre loi et le trafic d´organes

Nos législateurs n´ont jamais vu dans le commerce d´organes une source de greffons mais l´ont toujours condamné et considéré comme un crime, qui porte atteinte à la personne humaine et à sa dignité. Et à ce titre, envisager la pratique de la greffe rénale dans les cliniques chirurgicales privées, ne fait que basculer une partie des patients disposant d´un donneur du secteur public vers celui du prive´, sans pour autant apporter une quelconque solution pour les malheureux patients ne disposant d´aucun donneur, ces cliniques chirurgicales dans les pays qui pratiquent le tourisme de transplantation sont désignées comme étant l´élément clé, facilitateur du commerce d´organes [2, 3]. Pour toutes ces raisons nos législateurs ont conjugué aux dispositions punitives des dispositions préventives, parmi ces dernières il y´a celle n´autorisant cette activité que dans le secteur public [4].

### La hauteur et les limites de l´apport du donneur vivant

#### Dans les différentes régions du monde concernant la greffe rénale

En dehors des pays qui pratiquent le tourisme de transplantation à outrance usant de filières internationales suspectes et bien organisées, on constate dans les différentes régions du monde et notamment dans les pays où les lois de la bioéthique sont scrupuleusement respectées, que le don de rein à partir des vivants reste une source insuffisante, malgré l´acceptation de plus en plus fréquente par beaucoup de leurs centres, du don en incompatibilité ABO et du don non apparenté « altruiste et affectif »: 1) les USA, en 2017, leur activité avait atteint 43,6 greffes rénales pmh à partir de donneurs décèdes et 18,06 Tx rénale pmh à partir de greffons issus du don entre les vivants, dont seulement 12,2 pmh provenaient d´un don apparenté [5, 6]. 2) En Europe: sous l´égide de l´UE [7], un rapport de 2016, avait montré que la moyenne des greffes rénales réalisées à partir des donneurs vivants pmh par les 28 états membres de cette union, était de 8,4 pmh. Et en analysant cette source de don par pays, on constate que: 14 pays (soit la moitié) réalisaient moins de 5 greffes pmh; 9 pays (le tiers) entre 5-15 greffes pmh et 5 pays seulement (soit 1,8% de l´ensemble de ces pays) plus de 15 greffes pmh. 3) En Asie: le Japon, où les greffons sont dans 90% des cas issus du don vivant pour des raisons culturelles connues, n´a réalisé´ en 2015 à partir de ce type de don que 11,75 greffes rénales pmh et si on enlève les 30% réalisées en incompatibilité ABO, ce taux serait de 8,22 greffes pmh [8].

La Corée du Sud a réalisé 24,33 greffes à partir du don vivant pmh en 2017 [5], un pays dont le tiers [9] de cette activité est réalisé en incompatibilité ABO. En ôtant cette modalité, on sera à 16,2 greffes pmh, sachant que dans ce taux, il y a des greffes réalisées à partir de donneurs non apparentés. Et en 2018, la Chine en a réalisé 1.8 pmh à partir de donneurs vivants, pour une population de 1.39 milliard et l´Inde en 2019, juste cinq greffes pmh pour une population de 1,35 milliard.

Concernant les pays du Moyen-Orient du groupe MESOT (Middle East Society for Organ Transplantation), un document [10] avait colligé l´ensemble de leurs résultats annuels de 2013, ce recueil de résultats permettant d´atténuer relativement le biais de certains de leurs pays qui pratiquent le tourisme de transplantation, a montré que la moyenne de leurs activités regroupées ne dépassait pas les 11,5 transplantations rénales pmh et dont seulement 8 greffes pmh étaient issues des donneurs vivants.

Finalement, si on tient compte uniquement de cette source de don, celle des vivants et si on ôte le don non apparenté et les transplantations en incompatibilité ABO, ce taux de 7 pmh réalisé actuellement chez nous n´est pas loin de la moyenne des greffes de 8,4 pmh des pays de l´UE réunis, celle des pays du Moyen-Orient groupés et celle du Japon et tout en étant très au-dessus de ce que réalise la Chine et l´Inde, deux pays où on peut espérer recruter le plus de donneurs vivants.

En conséquence, il est tout à fait illusoire de croire que notre pays pourrait atteindre le minimum de 35 pmh uniquement avec cette source, juste en développant les autres formes de ce type de don « le don croisé et l´ABO incompatible » ou en élargissant encore plus le cercle des donneurs vivants « le don altruiste ».

### Le don de rein issu des vivants en Algérie: perspectives et limites

Certes, concernant le don entre les vivants, un pays comme le nôtre, disposant de 13 centres accrédités à cette activité, ne peut s´auto satisfaire de 7 Tx pmh, raison pour laquelle une des recommandations phare, ayant été´ approuvée par l´ensemble des experts de la transplantation rénale des trois pays du Maghreb « la Tunisie le Maroc et l´Algérie », présents au 6e`me colloque France Maghreb sur la Transplantation, le 09 et 10 janvier 2015 à Fès, est que chacun de leurs centres doit atteindre un objectif annuel de 50 greffes rénale [11] dans un délais de 5 ans, ce qui fait pour notre pays, un total de 650 greffes /an, soit « 15 greffes pmh ».

Nos 13 centres, pour pouvoir s´aligner sur ce seuil minimal Maghrébin espéré, celui de 50 greffes/an/centre, à partir uniquement de cette source, leurs équipes doivent aussi puiser dans les autres formes autorisées du don entre les vivants, mais non encore développées, notamment et idéalement le don croisé pour ses avantages sus cités et à défaut celui en incompatibilité ABO.

Et même atteint, ce ratio de 15 greffes pmh reste très en deçà des espérances de nos malades en dialyse et des attentes de nos autorités sanitaires pour régler ce problème de sante´ public.

### Le recours au donneur décédé: une priorité

#### Des malades potentiellement curables mais condamnés à mourir

Pour toutes ces raisons, le recours au donneur décédé devient plus qu´une exigence et aussi parce que cette autre source de don reste l´unique solution salvatrice de ceux ne disposant d´aucun donneur vivant et surtout de ceux qui attendent un organe vital: cœur, poumon, foie, ou un rein pour ceux n´ayant plus la possibilité de faire la dialyse; des patients condamnés à mourir s´ils ne sont pas transplantés dans les semaines à venir. Encore plus tragique: ces patients à la fois très graves et potentiellement curables, n´auront jamais cette chance, dont dispose certains de nos malades des autres spécialités, celle d´être proposé à une prise en charge pour soins à l´étranger.

Les responsables de ces pays étrangers sont conscients que quel que soit le niveau maximal de leur activité, il y´aurait toujours un taux incompressible de leurs patients en liste d´attente qui vont décéder suite à un délai d´attente trop long, un délai insupportable pour leurs concitoyens malades, une situation générée par l´inadéquation universelle entre les greffons disponibles et le nombre de patient inscrits sur cette liste.

Il est donc tout à fait compréhensible que les responsables de ces pays étrangers ne puissent se permettre de rajouter sur leurs longues listes d´attente de greffe, des patients d´un autre pays, quel que soit la hauteur de la rétribution financière proposée par cet autre pays.

#### Une source inépuisable aux bénéfices certains pour un faible coût

Aussi il faut rappeler que cette source de don, la seule pour le sauvetage de cette catégorie de patients en insuffisance terminale d´un organe vital sans aucune possibilité d´un autre traitement de suppléance, permet une thérapeutique de choix, qu´est la transplantation, avec des taux de survie inespérés à 5, 10 ans et plus, des taux dépassant de loin ceux d´autres pathologies aussi graves, pour lesquelles et de façon très justifiée, beaucoup d´infrastructures et de centres ont été installés et des moyens thérapeutiques très couteux leurs ont été consacrés.

Alors que le coût du séjour en réanimation, de ces potentiels donneurs, une fois en état de mort encéphalique, qui d´ailleurs est très court, parait dérisoire devant les moyens thérapeutiques budgétivores mis à disposition de ces autres pathologies aussi graves. Ces organes, qui pour leurs maintenir un état hémodynamique correcte chez ce donneur décédé, durant quelques heures, n´ont pas besoin de moyens supplémentaires par rapport à ce qui était déjà disponible au niveau de cette même réanimation et mis à disposition de la prise en charge de ce même donneur avant qu´ils ne décèdent.

Également l´activité de prélèvement de ces organes et contrairement aux autres activités chirurgicales n´a besoin ni d´un centre ni d´un service de chirurgie dédiés et encore moins d´une salle opératoire dédiée, une activité qui a besoin d´emprunter, juste pendant la durée du prélèvement, une des salles opératoires dédiés à ces autres activités, pourvue qu´elle soit vaste et à proximité de ce service de réanimation. En plus, à part la solution de conservation réfrigérée, qui est spécifique à cette activité, le reste des moyens chirurgicaux nécessaires à ce prélèvement est le standard déjà existant, au niveau de cette même salle opératoire, utilisé pour une chirurgie générale, vasculaire ou urologique.

Concernant cet aspect coût: la décence aurait voulu, qu´on ne devrait même pas l´évoquer ni celui des économies rapportées par ce même programme par rapport à celui de la dialyse, car pour ces patients qui ne peuvent bénéficier d´un traitement de suppléance autre que la greffe, il s´agit de vie ou de mort et la vie humaine n´a pas de prix. Mais si on a abordé cet aspect financier, c´est surtout pour souligner qu´il n´est pas un frein, mais plutôt une incitation à développer ce type de don d´organes.

#### Nos dispositions réglementaires régissant ce type de don et la position de la religion

Face à cette catégorie de malades graves en attente d´une transplantation salvatrice: le don d´organes à partir de nos concitoyens décédés en état de mort encéphalique a été élevés au premier plan des préoccupations de nos autorités sanitaires, à travers notre loi de santé de 2018, qui a posé les jalons pratiques et éthiques indispensables à la concrétisation et à l´aboutissement en toute sécurité de cette activité, une loi dont les décrets nécessaires et relatifs à son application seront promulgués dans un délai que nous espérons très proche. En plus de cette nouvelle loi de bioéthique [4], à la fois structurante pour les équipes en charge de cette activité et très favorable aux prélèvements d´organes sur donneur décédés, la religion majoritaire, dans ce pays, non seulement ne s´oppose pas à ce type de don, mais elle le considère comme un acte de charité « une aumône continue » [12, 13].

#### Les grandes lignes d´un programme à donneurs décédés réussi

À ce titre, il est important de rappeler, que les pays ayant su se servir de cette source de don à la fois inépuisable et non suffisante, tout un programme national lui a été consacré, priorisé et développé, un programme aussi vital pour leurs malades graves que délicat et intrusif voir violent pour des familles qui viennent d´être endeuillées par la perte d´un être cher, puisque touchant à l'intégrité du corps de leur défunt « ce donneur décédé » et à sa sacralité.

En substance, les autorités sanitaires de ces pays, en étant très conscientes des contraintes émotionnelles insoutenables de la part des familles des donneurs, des considérations éthiques liées à ce type d´activité et des exigences professionnelles et organisationnelles nécessaires à la réussite d´un programme dont les résultats ne sont plus à démontrer: se sont appuyés, aux niveaux de leurs hôpitaux, sur quelques centaines de personnes recrutées parmi leurs professionnels de santé public, médecins et infirmiers, aux profils bien choisis et formés spécifiquement à la coordination intra et extra hospitalière de prélèvement d´organes et surtout au dépistage des potentiels donneurs décédés et à l´abord de leurs proches, un abord rendu facile par la qualité de la relation qu´entretenait le personnel soignant avec la famille du défunt avant son décès.

Ce personnel, formé à ces trois missions clés, des missions ne tolérant aucune forme d´amateurisme ou d´improvisation, est directement supervisé par leurs agences de greffe, affecté et déployé en réseaux « locaux, régionaux et national », couvrant l´ensemble des lits de réanimation de tous leurs territoires nationaux, avec une permanence disponible 24 heures/24 et 7 jours/7.

Une activité qui exige de ce personnel une grande vigilance quant aux critères validant la prélevabilité des organes de ces potentiels donneurs dépistés et une attention particulière aux normes de sécurité sanitaire pour éviter tout risque de transmission de pathologies infectieuses ou néoplasiques à ces candidats à la greffe. Ces normes et ces critères sont des mesures protectrices pour les futurs receveurs.

Et pour éviter tout conflit de missions, il a été imposé aux équipes de réanimation en charge de ces potentiels donneurs et éventuellement de leurs constats de décès de ne pas faire partie des équipes médico-chirurgicales en charge de la transplantation et réciproquement; deux responsabilités qui peuvent entrer en conflit si elles sont exercées simultanément par une même équipe, une mesure protectrice de ce potentiel donneur avant qu´il ne décède, au même titre que celle qui exige à ces mêmes équipes de réanimation que le diagnostic clinique de la mort encéphalique soit documenté médico-légalement par un examen para clinique « l´EEG ou par un angioscanner cérébral ».

Ce type de don, fait aussi craindre certaines dérives immorale ou pathologique en post don, comme un chantage financier de la part de la famille du donneur décédé ou d´un de ses membres, ou aussi le développement d´une relation affective pathologique entre un parent proche du défunt et celui qui porte l´organe de son parent « le receveur », soulevant ainsi de vraies inquiétudes et dont la réponse a été apportée par l´anonymisation du donneur décédé, une autre mesure protectrice concernant les éventuels receveurs.

Et pour s´assurer à la fois de cet anonymat du donneur décédé et d´une attribution qui soit la plus transparente et la plus juste de ces organes à usage thérapeutique, ces pays se sont appuyés, mais cette fois-ci au niveau de leurs agences de greffe, sur des techniciens et des gestionnaires ayant une maitrise parfaite des tenants et aboutissants de cette activité, un personnel comme celui de la coordination de prélèvement aux niveaux des hôpitaux, qui soit dépourvu de tout lien d´intérêt avec les centres de greffe de leurs pays.

Pour ce faire, ces agences se sont également dotées d´une liste d´attente nationale informatisée et actualisée de tous leurs candidats inscrits à la greffe, une liste alimentée et consultable par leurs équipes de transplantations et d´un système informatique national d´attribution de ces greffons, une attribution se basant principalement sur des critères d´équité et d´efficacité convertis en score, un scoring qui aussi prend en considération les cas prioritaires et les contraintes techniques liées au transport et au maintien de la qualité de ces greffons.

Un système informatique d´attribution perfectible, puisque réévalué périodiquement et auquel des modifications sont apportées régulièrement en faveur des catégories de candidats ayant été défavorisés antérieurement. Une stratégie capitale à ces agences pour qu´elles puissent assoir leur rôle de régulation.

Parmi les autres rôles déterminants de cette coordination de prélèvement d´organes celui de veiller sur trois taches qui doivent être exécutées dans des délais à ne pas dépasser: celle du transport des équipes en charge de ces prélèvements compte tenu de la courte phase à cœur battant du donneur en état de mort encéphalique, celle des conditions de conservation des greffons et de leurs transports et celle de l´acheminement des prélèvements sanguins et tissulaires nécessaires aux tests d´histocompatibilité, des tests indispensables à l´attributions de certains organes.

Un programme dont l´application relève de l´entière et l´unique responsabilité de leurs agences de greffe, avec une multitude de taches qui ne peuvent être fondées sur le seul engagement de chefs de services hospitaliers ou hospitalo-universitaires ou des directeurs de leurs établissements.

Une organisation et un personnel dont le professionnalisme et l´entière disponibilité, ont réussi au bout de quelques années, à réduire graduellement le taux d´opposition à ce type de don à 30 et à 14%, chez deux pays proches géographiquement de nos pays du Maghreb et surtout à balayer définitivement des préjugés autrefois véhiculés par le manque de formation, l´insuffisance de connaissances et les tergiversions. Des fausses croyances qui accablaient systématiquement et injustement l´implication de leurs sociétés vis-à-vis de ce type de don, en invoquant à tort: tantôt la religion, tantôt le manque de générosité et tantôt les appartenances ethniques ou culturelles de leurs concitoyens.

Une organisation et un savoir-faire qui ont su mettre au profit de ce type de don salvateur les spécificités de leurs systèmes de santé et les caractéristiques socioculturelles et religieuses de leurs populations. Et afin d´optimiser au mieux cette source de don beaucoup de ces pays ont opté pour le consentement implicite, où tous leurs citoyens sont présumés donneurs d'organes après leurs décès, sauf ceux ayant exprimé de leur vivant soit par écrit soit oralement, leurs refus d´être prélevé.

Un programme dont le haut sens de l´éthique et la grande rigueur dans sa mise en application, ont permis au personnel de coordination de prélèvement d´organes de ces deux pays, d´éviter d´essuyer une opposition exprimée, au nom du défunt, par les familles de 1 871 donneurs décédés prélevés, contre 802 oppositions, pour l´un, et 2 295 donneurs décédés prélevés contre seulement 360 oppositions pour l´autre, en 2019 [14].

Un programme avec d´excellents résultats à la clé, où pour chacun de ces deux pays: plus de 5 000 de leurs patients adultes et enfants bénéficient, chaque année, d´une transplantation d´organe, des transplantations qui sont au minimum salvatrices dans un cas sur trois, soit environ 1500 de leurs malades qui étaient condamnés à mourir se sont vus se soustraire de cette liste d´attente, une liste qualifiée par ces patients sauvés de couloir de la mort et qui sont, dans leur majorité, après être greffés, entrain de vaquer, à leurs occupations familiales et professionnelles.

A tous ce travail s´ajoute celui d´une stratégie efficiente de la communication associative et celle de leurs agences de greffe, concernant la sensibilisation du grand public, au don d´organes, notamment celui en post mortem, en usant de tous les supports pédagogiques et médiatiques nécessaires et adéquats.

Il faut aussi rappeler que pour ces pays ayant su se servir de cette autre source de don, la gestion de ces organes, relève de la souveraineté de leurs autorités sanitaires: tant que cette source de don demeure l´unique à permettre la seule modalité thérapeutique salvatrice pour ce type de malades graves et tant que ces greffons issus des donneurs décédés resteront insuffisants pour satisfaire les besoins de tous leurs patients. Des greffons qui, pour ces deux raisons rationnelles, resteront non partageables avec les malades des autres pays, notamment ceux non soucieux de développer ce type de don.

## Conclusion

Concernant nos malades ne disposant d´aucun donneur vivant et ceux en insuffisance terminale d´un organe vital, sans aucune possibilité d´un traitement de suppléance, le don d´organes entre les vivants, ne peut se substituer à celui issu des personnes décédées.

Pour ce type de patients graves et comme souligné en introduction, aucune donnée statistique, même estimée, concernant leur nombre, ne nous est disponible, le premier pas à réaliser dans ce programme salvateur, consiste à instaurer cette liste d´attente nationale informatisée et actualisée avec le système informatique d´attribution, deux outils fiables, les seuls à nous renseigner sur leur nombre annuel réel, sur le nombre de ceux devenant inéligibles à la transplantation suite à une dégradation sévère de leur état de santé et celui de ceux ayant décédé parmi ces inscrits sur cette même liste.

Des données statistiques instantanées et annuelles, d´une valeur capitale pour prendre conscience de ces décès, des besoins chiffrés nécessaires à cette activité « types et nombre de greffons » et surtout pour déterminer les responsabilités qui incombent à chacun.

Faire de cette activité de prélèvement d´organes sur personnes décédés une priorité nationale est une décision juste et inéluctable, pour répondre aux pressions énormes exercées sur nos équipes médicales en charge de ces malades graves, des besoins et des pressions qui ne cessent d´augmenter, y compris de la part des familles de ces patients.

Un programme dont les moyens et les compétences nécessaires sont à la portée de notre pays et à celle de beaucoup d´autres pays africains à revenu intermédiaire « à mon sens », à condition que cette activité soit fondée sur les mêmes normes d´éthique, de recrutement, de formation et organisationnelles, des autres pays qui ont acquis dans ce domaine un savoir-faire inestimable et des résultats excellents.
